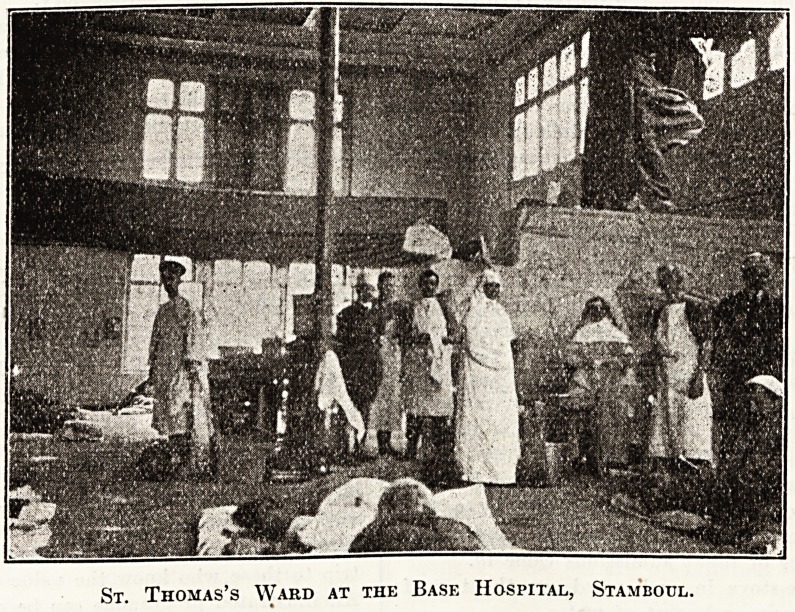# The Organisation of the British Red Cross Hospitals in Turkey

**Published:** 1913-03-15

**Authors:** C. M. Page

**Affiliations:** on the Staff of the Base Hospital at Stamboul.


### THE ORGANISATION OF THE BRITISH RED CROSS HOSPITALS IN TURKEY.

**By C. M. PAGE, M.S., F.R.C.S., on the Staff of the Base Hospital at Stamboul.**

It must always be a difficult task to constitute an effective voluntary aid detachment to such a country as Turkey in time of war. An accurate knowledge of the existent conditions and of the probable seat of operations cannot be obtained in time to be of assistance in the choice of the equipment and personnel. The distance from England is such that, unless unlimited funds are available, the provision of accommodation and transport must be left almost entirely to the local authorities.

Under these circumstances the British Red Cross Society decided to send to Turkey three units, each of eighteen men, under the charge of Major Doughty-Wylie. Each unit was furnished with the essential medical stores for a mobile party and a skeleton equipment for a small field hospital.

### Making a Museum into a Hospital.

Reinforcements, both of personnel and material, arrived in December, and ultimately three working hospitals were constituted—the base at Constantinople, one on Lake Kuchuk Chekmiji, and the last at Bakos for suspected cholera cases. I propose here to deal with the organisation of the base hospital in Stamboul.

At the time of our arrival (November 5), the rapid course of the war and the forced retreat of the Turks to within a short distance of the capital resulted in a great concentration of the sick and wounded; according to the general estimate, they numbered within the walls from 15,000 to 20,000, and there was only accommodation ready for some 4,000 or 5,000. Under these conditions the Ottoman Red Crescent Society handed over to us the building of the Musée des Beaux-Arts, which had been placed at their disposal, and cleared of statues by the director, Halil Bey. The Museum was a fairly recent structure, and although—in common with most other buildings in Stamboul—it had practically no sanitary accommodation, it stands on the side of a hill inside the Seraglio walls, with no inhabited houses near, and was backed by about half-an-acre of partly cultivated garden; it had the further advantage of being only five minutes' walk from the railway receiving station for the wounded.

### Arranging Wards and Quarters.

There are eleven rooms in the building available—nine on the main floor, and two in the basement quite separate from the others. The latter were employed for isolation purposes, and of the former, one, with a stone floor and good light, was converted into the operating theatre, one was used as a store-room, and at the commencement, four were taken up as quarters for the personnel, leaving the three largest for general wards. The total accommodation available for patients was about 100 beds. The flooring of the rooms, with one exception, was of smooth, unpolished boarding; this proved very difficult to keep clear of dust, especially while there were no bedsteads available; in December the ward floors were covered with a cheap oilcloth, which was much more easily kept clean. Ventilation was difficult to maintain, as only the windows on the ground level were constructed so as to open.

This building we occupied on November 6, and on the evening of the following day admitted some thirty cases of severely wounded; these were followed by a similar batch on the next day. It can well be imagined that the organisation to cope with the regular care of the patients was not completed at once, but the best idea of the arrangements will be given by dealing in turn with the several factors which had to be considered.

St. Thomas's Ward at the Base Hospital, Stamboul.

### Our Medical Equipment.

This consisted, for each unit, of the Army Medical Surgical and Medical Field Panniers, a reserve dressing-box containing extra chloroform, carbolic, and tincture of opium. Three fitted surgical haversacks and the medical companion (R.A.M.C. pattern) were also taken. A few extra instruments were taken by the medical officers. The outfit in general proved very satisfactory; a shortage of antiseptic solution was however soon felt. The chief form in which this had been carried was crystalline carbolic-acid—a very bulky method—pure lysol would be much more compact and gives an excellent solution. Crude phenol for disinfecting bed pans, etc., and chloride of lime for the latrines was very expensive, but a large present of both was sent out in December.

### Some Lessons on Hospital Equipment.

This primarily consisted of the R.A.M.C. Cavalry Field Ambulance Panniers, reinforced by some hundred extra bed pans and urinals. One hundred enamelled mugs and several hundred blankets and ground sheets were also taken; the latter were made of Willesden canvas, and proved very unsatisfactory for the many enteritis cases; the canvas was porous and extremely difficult to clean; stout jaconet would have been much better. Some cheap American cloth bought in Constantinople proved very useful for a time as a substitute.

A hundred camp-beds were presented by Mrs. Doughty-Wylie, but they did not arrive till late in December. Mattresses—wide and clumsy—were given by the Ottoman Red Crescent; the patients were very comfortable on these on the floor, but nursing and dressing were rendered very arduous. We had some simple cross-stretcher bedsteads made of hard wood and covered with canvas. These proved convenient for medical cases, but were hardly rigid enough for those of fracture.

Pillows, linen, and additional blankets were presented by the Ottoman Red Crescent Society.

### The Kitchen, Patients and "Sour Milk."

For the first week all cooking was carried out in camp kettles on a trench wood fire, or in small saucepans on Primus stoves. Subsequently we had two large stoves put in wood sheds—one for the patients, worked by their own cooks (essential for Mohammedans), and one for the personnel.

The cooking for the patients was of a simple nature—a mess of rice, meat, and vegetables being the staple of their diet. They drank tea readily, but were always suspicious of any soup or meat extract. They consumed nearly a pound of bread a day per man, and as a delicacy were given "jaodt"—the native curdled sour milk.

The supply of fresh meat and vegetables was maintained with moderate punctuality and at a fair cost. The most expensive item in the consumable stores was coal, or, rather, coke; this was always difficult to obtain and cost £4 a ton; charcoal was correspondingly dear.

### Water Supply.

The Museum authorities caused three taps connected with Derkos reservoir to be fitted soon after our arrival, but this water was not fitted for drinking; a daily supply of water for this purpose was brought in barrels from a spring; this water was filtered through canvas or a Berkefeld canister filter before use. A fair if somewhat uncertain supply of hot water for the wards was kept up by using charcoal-heated samovars.

### Light and Heat.

A gas-jet was installed in each room of the hospital within a week of our arrival. The lighting by a few lamps, at first necessary, was extremely inconvenient when a large number of night admissions came in.

There was a coke-stove in each ward, on the top of which was kept a basin or bucket of water.

### Sanitary Arrangements.

The Museum was fitted with three latrines of the native type, consisting simply of a small hole in a flagstone placed over the cesspool. These were very foul and so were shut up, and sets of bench latrines, with old kerosine tins as reservoirs, were put up in the grounds; here also urine, refuse, and a destructor pit were dug. The excreta from each ward were thrown into large, covered cans kept just outside; these were hourly emptied into pits by two natives, under the direction of the sanitary duty orderly.

Laundry work proved a continual difficulty; the native workers used much soap with slight and slow effect. In bad weather the drying of the linen was also rather a problem.

### Arrival of the Wounded.

The wounded often arrived in batches of ten or more; in turn they were placed on tables in the vestibule, their wounds inspected and any urgent treatment given. Their names were entered in a book, together with the diagnosis, and two labels with their name and hospital number written on—one for the man's kit, the other to hang over his bed. They were then removed to their ward, and there cleaned up on a ground sheet before being put in bed. Practically all kit was sent in separate sacks to be steam-sterilised by the local authorities.

### Orderlies and Nurses.

No female trained nurses were taken out with the detachment, but Mrs. Doughty-Wylie obtained the services of two French sisters, two Turkish ladies, and an Armenian nurse. For work at a base hospital the large number of English orderlies taken did not prove altogether satisfactory. They were not always contented with their work, and their behaviour in the town often left much to be desired. The Turkish servants who were hired to do ward work were extremely useful—and cheap.

In my opinion the most satisfactory equipment, as far as personnel is concerned, for a similar expedition would be several picked trained female nurses, a similar number of first-class male nurses, a cook, and a carpenter. For general work and rough nursing the natives, under supervision, are quite satisfactory. A reliable ex-N.C.O. would be valuable as a quartermaster.

It is, of course, easy to offer advice after the event, and in choosing personnel for a Red Cross Mission the uncertainty of the conditions to be dealt with must always render the achievement of the ideal a matter of chance.

## Figures and Tables

**Figure f1:**